# Delayed Presentation of a Postoperative Biloma Requiring Multimodal Intervention in an Elderly Female: A Case Report

**DOI:** 10.7759/cureus.91858

**Published:** 2025-09-08

**Authors:** Se Jong Choi, Veronica Rasmusen, Niki Mohammadi

**Affiliations:** 1 Internal Medicine, California University of Science and Medicine, Arrowhead Regional Medical Center, Colton, USA; 2 Internal Medicine, Arrowhead Regional Medical Center, Colton, USA

**Keywords:** bile leak, biloma, cholecystectomy, elderly, ercp, general surgery, interventional radiology, patient-doctor communication, sepsis

## Abstract

Biloma is a rare but serious postoperative complication, often resulting from iatrogenic bile duct injury. We present the case of an 81-year-old woman with a delayed presentation of biloma complicated by sepsis, who required both interventional radiology (IR)-guided drainage and endoscopic retrograde cholangiopancreatography (ERCP) with biliary stenting for management. There was a delay in diagnosis and management for this case, particularly due to a combination of subtle clinical signs, language and hearing barriers, and limited patient understanding of management. This report emphasizes the importance of multidisciplinary care, culturally competent communication, and flexibility in managing bile leaks in older adults.

## Introduction

Biloma, by definition, is an encapsulated collection of bile outside the biliary tree, mainly caused by trauma or iatrogenic injury. Most iatrogenic causes include laparoscopic cholecystectomy due to cystic duct stump leaks, accessory duct injury, or, less commonly, major bile duct injury [1-3]. The incidence of bile leaks after cholecystectomy is 0.4-1.1%, with biloma representing a subset of these complications [1,2,4].

Older adults and those with hearing or language barriers may experience delays in diagnosis due to atypical symptoms or communication difficulties [4]. Common presenting signs include abdominal pain, fever, or unexplained sepsis. While abdominal ultrasound or CT may reveal fluid collections, they do not confirm bile as the fluid source [3,5-7]. Hepatobiliary scintigraphy (HIDA), magnetic resonance cholangiopancreatography (MRCP), or endoscopic retrograde cholangiopancreatography (ERCP) is often required for a definitive diagnosis [8-11].

Management typically involves percutaneous drainage to relieve symptoms and reduce sepsis risk. ERCP with biliary stenting or sphincterotomy is often performed to divert bile flow and facilitate healing [1,2,7]. International guidelines, such as those from the World Society of Emergency Surgery (WSES), recommend early multidisciplinary involvement and broad-spectrum antibiotics for patients with suspected bile duct injury [10]. Surgery is reserved for refractory or complex cases [4]. In selected cases, advanced interventional radiology (IR) techniques, such as coil embolization of the cystic duct stump, may be employed [12]. Timely, multidisciplinary collaboration is essential to ensure appropriate intervention, particularly in high-risk populations [5,6].

## Case presentation

An 81-year-old, Spanish-speaking female with a history of type 2 diabetes, hypothyroidism, and hyperlipidemia underwent an elective laparoscopic cholecystectomy for symptomatic cholelithiasis at an outside hospital. Her postoperative course was complicated by postoperative biliary sepsis requiring intravenous broad-spectrum antibiotics and prolonged hospitalization. She was discharged on postoperative day 12 with a plan for continued intravenous antibiotics due to persistent concern for intra-abdominal infection.

**Figure 1:**
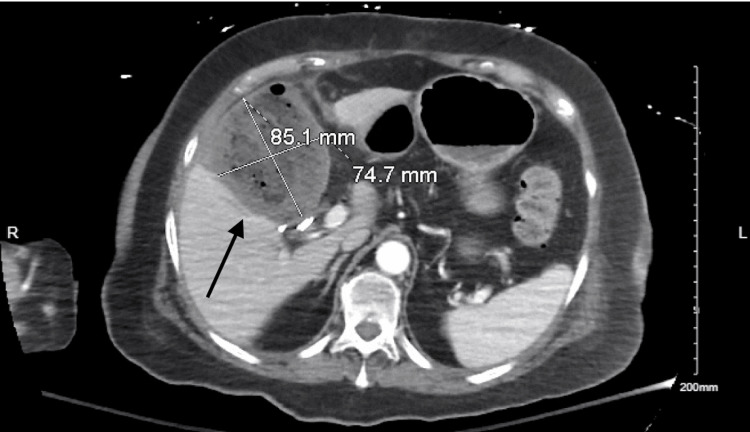
Contrast-enhanced CT scan revealing a large biloma in the subhepatic space

**Figure 2:**
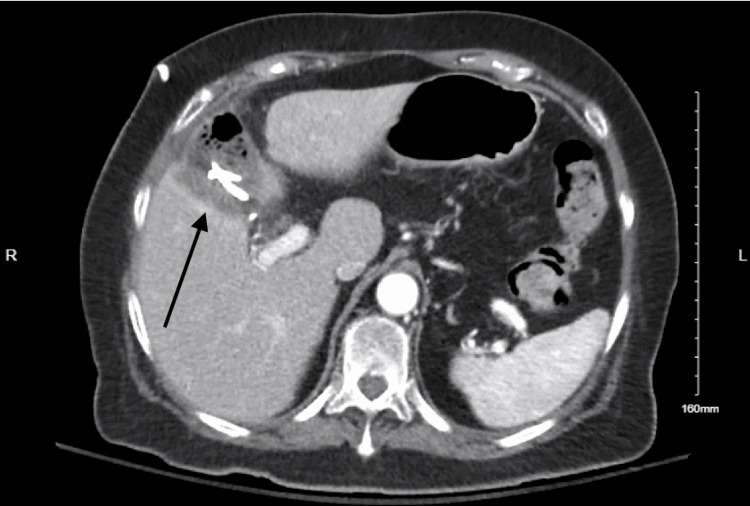
Contrast-enhanced CT scan with a drain in place, showing perihepatic fluid collection

She re-presented three days later with abdominal pain, nausea, and dyspnea. Vital signs showed hypertension (systolic 140s) and oxygen saturation in the low 90s on room air. Labs revealed leukocytosis (WBC 12.3), anemia (Hgb 10.7), and neutrophilia. Liver function tests were within normal limits, and CRP was not obtained at that time. Contrast-enhanced CT demonstrated a 7.6 × 7.5 × 8.5 cm fluid collection in the gallbladder fossa, consistent with a biloma (Figure 1). She was diagnosed with sepsis secondary to a postoperative intra-abdominal abscess. Interventional radiology performed image-guided drainage, and a subsequent CT confirmed catheter placement with residual perihepatic fluid (Figure 2). The drain produced bilious output, raising concern for a bile leak.

**Figure 3:**
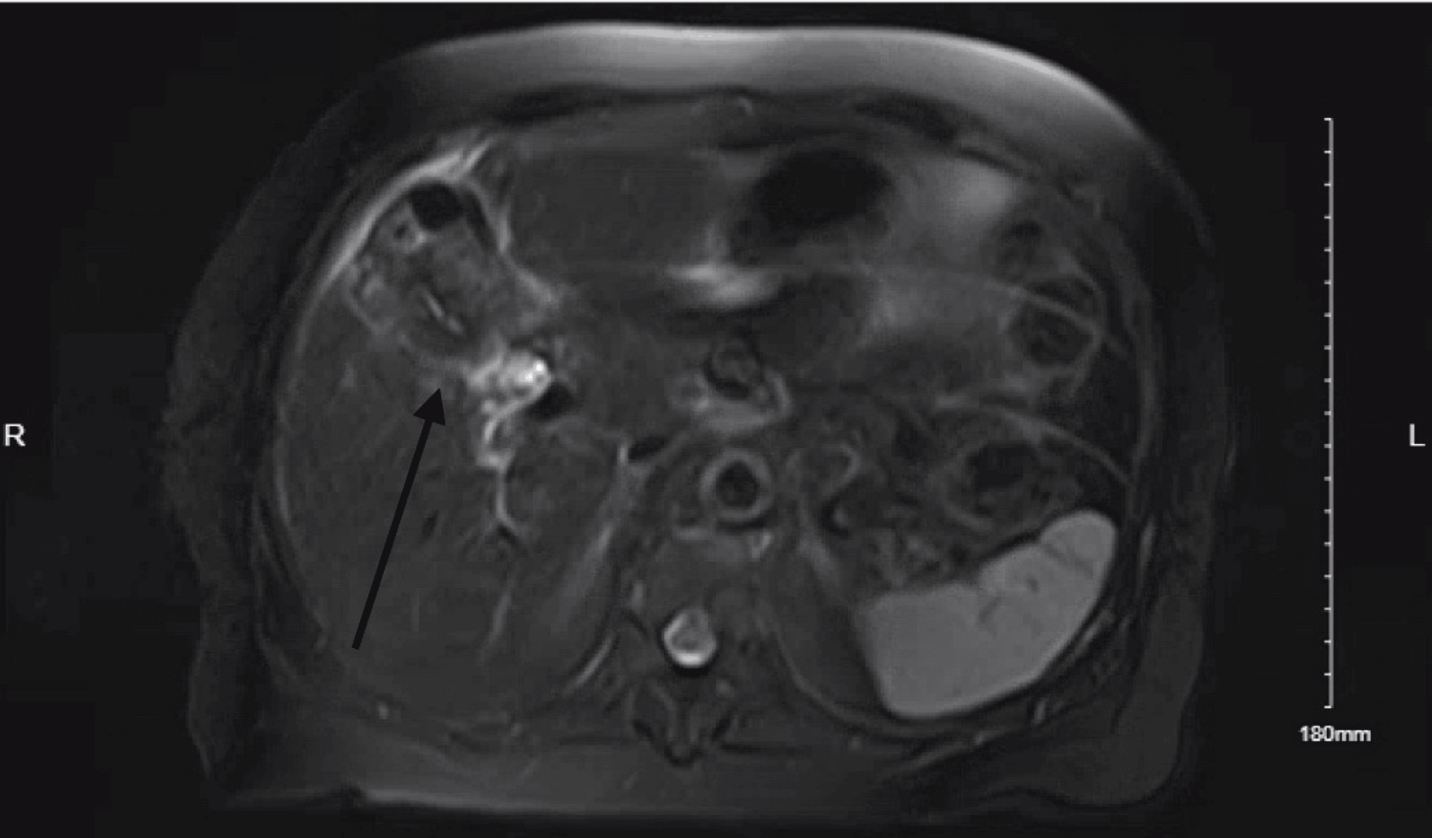
MRCP image showing a loculated fluid collection in the gallbladder fossa, suggestive of a biloma MRCP: magnetic resonance cholangiopancreatography

Despite initial improvement, bilious output persisted. MRCP was obtained to further characterize the leak and demonstrated a complex, loculated fluid collection in the gallbladder fossa with mild biliary dilation, highly suggestive of a biloma (Figure 3). ERCP was recommended; however, the patient initially declined. She expressed uncertainty regarding the evolving care plan, as she had previously understood that antibiotics alone would suffice, leading to confusion.

Over the next several days, the multidisciplinary care team, including primary medicine, gastroenterology, general surgery, and IR conducted repeated sessions using both phone and in-person Spanish interpretation to clarify the rationale for intervention. The patient initially agreed to ERCP, then reconsidered and preferred antibiotics alone, ultimately consenting only to IR drainage. As bilious drainage persisted, the IR team upsized the drain. Microbiology from the drain fluid grew mixed enteric flora, guiding continuation of cefepime and metronidazole. Eventually, after consolidated counseling, she consented to ERCP.

**Figure 4:**
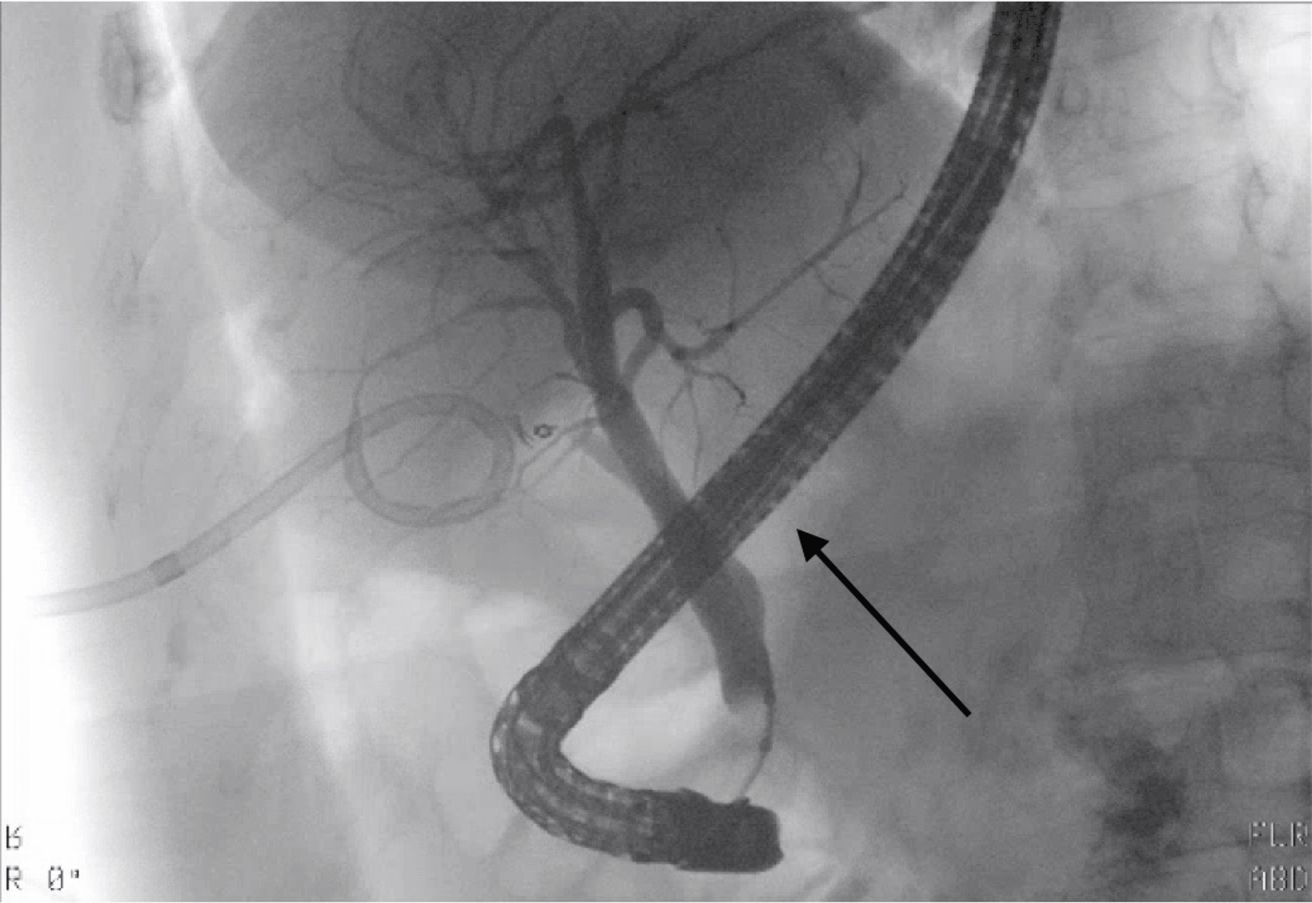
ERCP fluoroscopy image demonstrating biliary stent placement in the absence of overt contrast extravasation ERCP: endoscopic retrograde cholangiopancreatography

ERCP demonstrated no overt extravasation; however, a 10Fr × 7cm plastic biliary stent was placed empirically in the common bile duct to divert bile flow (Figure 4). Drain output subsequently decreased, and her symptoms resolved. She completed a 14-day antibiotic course tailored to culture results and was discharged to a skilled nursing facility for ongoing IV antibiotics, drain care, and rehabilitation, given her frailty and functional decline after hospitalization.

Her discharge plan included outpatient follow-up with gastroenterology, with repeat ERCP and stent removal scheduled at eight weeks.

## Discussion

This case illustrates several key challenges in diagnosing and managing biloma in older adults. The patient’s delayed presentation with vague symptoms highlights the importance of maintaining a high index of suspicion. Older adults with sensory or language limitations may underreport or misinterpret symptoms, increasing diagnostic complexity [4,8]. Importantly, delayed recognition of bile leaks can lead to sepsis, which is associated with increased morbidity and mortality in this population.

Imaging modalities, such as ultrasound or CT, can suggest biloma, but confirming bile as the source requires functional or anatomic imaging (e.g., HIDA) or ERCP/MRCP [1,3,5,9,11,13]. Recent reviews emphasize a multimodal imaging approach to reduce missed diagnoses [13]. In this case, MRCP helped delineate the fluid collection and raised suspicion for bile leakage, which was later addressed through ERCP-guided stenting [1,2,7].

From a management standpoint, the World Society of Emergency Surgery (WSES) guidelines recommend early recognition, antibiotics, and multidisciplinary involvement [10]. ERCP with biliary stenting remains the cornerstone of therapy. In cases where bile leaks are refractory, advanced strategies, such as coil embolization of the cystic duct stump, may be employed [12]. In situations where a subtotal cholecystectomy has already been performed, further minimally invasive surgical or interventional approaches may be required to manage persistent bile leaks [14].

Patient understanding was also a critical factor in the timeline to intervention. Initial explanations differed from evolving recommendations, contributing to confusion and hesitation. This emphasizes how inconsistent communication can delay decision-making. Consolidation of information by a single representative ultimately minimized confusion and facilitated informed consent. This highlights the broader importance of structured communication in improving patient outcomes.

## Conclusions

Bilomas require timely diagnosis and intervention to avoid complications. This case demonstrates that delayed recognition may occur in elderly patients, particularly those with communication challenges, and that effective shared decision-making and multidisciplinary care are essential for achieving successful outcomes. As reinforced by WSES guidelines, optimal care requires collaboration between surgeons, gastroenterologists, radiologists, and interventional specialists. Novel therapies, such as coil embolization of cystic duct stumps, may further expand the armamentarium for difficult cases.

Finally, this case underscores the importance of clear and consistent communication between providers and patients. For medical trainees and young physicians, it serves as a reminder that technical expertise must be paired with strong communication skills to ensure patient understanding, build trust, and support effective, patient-centered care.
